# News and Notes

**Published:** 1900-07

**Authors:** 


### *NEWS AND NOTES.*

SUMMER COMPLAINT.

O summer time! whose humid breeze
Brings stiffest collars to their knees.
Ripe time of freckle and of tan,
Of chafing babe, dissolving man,
Mosquitoes, flies and bumblebees,
Of fruit-engendered "diarrhees,"
Of country boarding treacheries,
Of insects' madd'ning rataplan,
O summer time!

Take all thy flowery rhapsodies,
Give me instead a nipping freeze!
Oh! for the frothing growler can!
Oh! for a mouth Gargantuan!
To mitigate such woes as these,
O summer time! W.

---

THE *Memphis Lancet* and the *Memphis Monthly* have become consolidated under the title of the last mentioned journal.

---

DR. HENRY HICKS, of Wrightsville, Ga., died in Dublin, Ga., on June 25th last. He was in his sixty-seventh year, and was one of the most prominent and influential physicians in that portion of the State.

---

THE death is announced of Dr. Apostoli, of Paris, France, the real father of electro-therapeutic treatment of uterine fibroids. His clinic in Paris had a world-wide reputation, and his practice was said to be one of the wealthiest in that city.

---

DR. PAUL GIBIER, the director and founder of the Pasteur Institute in New York, died on June 9th from injuries received in a runaway accident. He was a tireless worker, and did much to advance the well-known method of Pasteur in treating hydrophobia.

---

THE managing committee of the Liverpool School of Tropical Diseases will shortly despatch an expedition to the Amazon to investigate the yellow fever. The expedition will proceed by way of Baltimore, where its members will confer with physicians at Johns Hopkins University.

---

A WOMAN in New Jersey has otained a verdict of $8,000 against the Pennsylvania Railroad Company for a dislocated kidney caused by jar in coupling a car upon which she was a passenger. Her husband is to receive $2,000 additional for the loss of her companionship.

---

CUSTOMER: "Give me ten cents worth of paregoric, please."

Druggist: "Yes, sir."

Customer (absent-mindedly): "How much is it?"

Druggist: "A quarter."—*Exchange.*

---

"HERE'S the clock-maker come to fix our sitting-room clock," said the funny man's wife; "won't you go up and get it for him?"

"Why, it isn't upstairs, is it?" replied he, lazily.

"Of course it is. Where did you think it was?"

"Oh, I thought it had run down."—*Philadelphia Press.*

---

OLD MED: "Well, old man, how'd you sleep last night? Follow my advice about counting up?"

New Med: Yes, indeed. Counted up to 18,000."

Old Med: "Bully! And then you fell asleep, eh?"

New Med: "Guess not! It was morning by that time, and I had to get up."—*Pennsylvania Punch Bowl.*

THE ROSE AND THE THORN.—Servant (radiant)—Your first patient is waiting outside, sir.

Doctor—One? How is that? I thought I heard two men coming in.

Servant—The second was the landlord with his bill. He's been waiting for the patient.—*N. Y. World.*

---

MUSIC APPRECIATED.—Miss Thumpp-Hardie: "Did you ask Mrs. Nexdoor if my piano-playing disturbed her baby?"

Servant: "Yes, mum; and she said the baby liked it, and she was much obliged to you fer playin' so much."

Miss Thumpp-Hardie: "Did she, really?"

Servant: "Yes, mum. She said it saved her th' trouble of poundin' on a tin pan."—*N. Y. Weekly.*

---

THE opinion is gaining ground in the professon that the continued prevalence of grip is due very largely to the germs being carried into the houses by the trailing skirts of women. Already it has been recognized that the dried sputum of the tuberculous patients is being carried into homes and disseminated in the same way. Reform in this matter must come from the intelligent classes in every community. A few women of recognized social position could easily set the stamp of fashion upon the hygienic short walking skirt.

---

ON Sunday, May 6th, Dr. Jacobi was presented with a silver tankard, on which was engraved an inscription reading: "To Dr. Abraham Jacobi, on the seventieth anniversary of his birthday, from the Mount Sinai Hospital, in grateful recognition of forty years of devotion and fidelity, May 6, 1900." The ceremony took place at Mount Sinai, in the presence of the Medical Board and the Managers, and the presentation address was made by Isaac

Wallach, President of the Board of Managers. The tankard is nearly two feet high, and is decorated with the figure of a child touching the rod of Esculapius, and other designs emblematic of Dr. Jacobi's life-work in the field of disease of children.

---

IT is now nearly two years ago that attention was called to a new drug, named heroin, which was said to exert a specific action upon the respiratory organs, differing in many respects from the remedies previously in use. Careful experiments on animals and in clinical practice showed that heroin reduced the number of respirations while increasing their force, and also exerted a soothing influence upon the broncho-pulmonary tract, as evidenced by the relief of cough and dyspnea. The numerous clinical contributions that followed the first reports fully confirmed the earlier observations, except that they showed that the effects of heroin might be obtained with much smaller doses than were at first employed. In view of the fact, however, that large doses are still in use by many practitioners, it is surprising that after-effects have been so rarely observed, and these have consisted chiefly of slight lassitude and constipation; only four instances are on record in which they were considered of sufficient importance to be deemed worthy of mention, and even here they promptly subsided on discontinuing the drug. When compared with morphine and other opium derivatives the value of heroin becomes still more obvious. It is a matter of common observation that morphine is badly tolerated by many patients, and, according to such authorities as the United States Dispensatory, codein is often an uncertain drug. On the other hand, heroin acts efficiently and reliably in much smaller doses, and, owing to its freedom from any depressing action upon the heart, it can be utilized in many instances in which opiates would be contraindicated.

